# A Veil of Ignorance

**DOI:** 10.1017/S1478951526102831

**Published:** 2026-06-04

**Authors:** Justin Cordova

**Affiliations:** 1 Department of Anesthesia, Walter Reed National Military Medical Center, Bethesda, MD, USA; 2 Department of Anesthesia, Uniformed Services University, Bethesda, MD, USA

## A veil of ignorance

I am tempted by a veil of bliss-
ful ignorance.
I often spend days,

months, entire seasons,
wishing to be cradled
within its unknowing arms.

I yearn to revel
within its grasp, to be un-
altered, to be un-

blemished; I wish to be left un-
aware of pain and prognoses,
oblivious of the fact

that my patients are dying,
will die soon,
have died.

Most go slowly, fade quietly,
exit stage peacefully,
though some will not;

for some it is a frantic, phrenetic,
faltering rescue from the moment we begin.
Too often,

I pull back the curtain
and meet another dying patient ―
a hostage held by an unassailable enemy ―

and want simply to vanish.
I find myself longing
not to ask, begging

not to know, about
the daughters she’ll leave behind,
the job he worked so hard to get,

the life she desperately intends to live.
My soul aches
a little more each time

that his toddler is the same age as mine,
that her son is just learning to drive,
that he married his Juliet

just three short months ago.
These memories, this minutiae,
form echoes in the cave of my heart,

epitaphs engraved there
when these endings
have ended.

They are testaments of defeat
and desperation, of relapse
eclipsing remission,

but I cannot let them define the darkness.
I must see them brightly,
beautifully, as its contrast; they are

foils to the specter of death,
penumbra at the edge of its shadow.
Though I am tempted ―

perpetually, shamefully ―
by a blinding veil of ignorance, I must see
it be torn in two ―

daily, painfully ―
as I prepare my heart
to be etched anew.

